# A Cytosine Methyltransferase Homologue Is Essential for Sexual Development in *Aspergillus nidulans*

**DOI:** 10.1371/journal.pone.0002531

**Published:** 2008-06-25

**Authors:** Dong W. Lee, Michael Freitag, Eric U. Selker, Rodolfo Aramayo

**Affiliations:** 1 Department of Biology, College of Science, Texas A&M University, College Station, Texas, United States of America; 2 Department of Biology, Institute of Molecular Biology, University of Oregon, Eugene, Oregon, United States of America; University of Sevilla, Spain

## Abstract

**Background:**

The genome defense processes RIP (repeat-induced point mutation) in the filamentous fungus *Neurospora crassa*, and MIP (methylation induced premeiotically) in the fungus *Ascobolus immersus* depend on proteins with DNA methyltransferase (DMT) domains. Nevertheless, these proteins, RID and Masc1, respectively, have not been demonstrated to have DMT activity. We discovered a close homologue in *Aspergillus nidulans*, a fungus thought to have no methylation and no genome defense system comparable to RIP or MIP.

**Principal Findings:**

We report the cloning and characterization of the *DNA methyltransferase homologue A* (*dmtA*) gene from *Aspergillus nidulans*. We found that the *dmtA* locus encodes both a sense (*dmtA*) and an anti-sense transcript (*tmdA*). Both transcripts are expressed in vegetative, conidial and sexual tissues. We determined that *dmtA*, but not *tmdA*, is required for early sexual development and formation of viable ascospores. We also tested if DNA methylation accumulated in any of the *dmtA/tmdA* mutants we constructed, and found that in both asexual and sexual tissues, these mutants, just like wild-type strains, appear devoid of DNA methylation.

**Conclusions/Significance:**

Our results demonstrate that a DMT homologue closely related to proteins implicated in RIP and MIP has an essential developmental function in a fungus that appears to lack both DNA methylation and RIP or MIP. It remains formally possible that DmtA is a bona fide DMT, responsible for trace, undetected DNA methylation that is restricted to a few cells or transient but our work supports the idea that the DMT domain present in the RID/Masc1/DmtA family has a previously undescribed function.

## Introduction

DNA methylation is essential for normal development and differentiation of plants and mammals [1]–[11]. Fungi like *Neurospora crassa* show substantial DNA methylation despite the fact that the process in this organism is dispensable [12], [13]. In contrast, several well-studied organisms completely lack DNA methylation (e.g., *Saccharomyces cerevisiae*, *Schizosaccharomyces pombe* and *Caenorhabditis elegans*), while in others (e.g., *Drosophila melanogaster* and *Aspergillus flavus*) very little methylation has been reported [14]–[18], and its significance remains controversial [19].

Although extensively studied, much remains to be learned about the biology and relationship of eukaryotic DNA methyltransferases (DMTs). Putative and established eukaryotic DMTs can be separated into five families [11], [20]: 1) the DNMT1 or “maintenance DMT” family (e.g., mammalian Dnmt1 and plant MET1); 2) the DNMT3 or “*de novo* DMT” family (e.g., mammalian Dnmt3A and Dnmt3B and the plant DRMs) 3) the plant-specific chromomethylase (CMT) family (e.g., Arabidopsis CMT3); 4) the fungal-specific DMT-like family (e.g., *Ascobolus immersus* Masc1 and *Neurospora* RID); and 5) the DNMT2 family. Some fungal DMTs, like *Neurospora* DIM-2 and *Ascobolus* Masc2, do not fit well into any one of the above-mentioned groups, but are regarded as highly divergent members of the DNMT1 family; alternatively, they may constitute a sixth, fungal-specific family [11].

It is important to note that proteins that are similar to DMTs in their primary structure (i.e., putative DMTs) may not be true DMTs. This appears to be the case for members of the DNMT2 family that have been recently demonstrated to have tRNA^ASP^-methylation activity [19]. Similarly, DMTase activity has not been demonstrated for either RID [20] or Masc1 [21], putative DMTs associated with RIP (Repeat-Induced Point Mutation) in *Neurospora* and the related process, MIP (Methylation Induced Premeiotically), in *Ascobolus*, respectively. RIP, the first eukaryotic genome defense system discovered [22]–[26] alters DNA duplications during the sexual phase of the life cycle by introducing C:G to T:A transition mutations in both copies of the duplication [27], [28]. DNA sequences that have been subjected to RIP are usually, but not invariably, methylated [23]–[25]. The precise relationship between RIP and DNA methylation remains unknown, but it seems likely that RID is responsible for cytosine methylation and/or deamination, resulting in the observed transition mutations [20], [26], [29]–[31]. RIP is abolished in homozygous crosses with *rid* mutants [20].

As in *Neurospora*, the haploid parental genomes of *A. immersus* are scanned for DNA duplications after fertilization but before karyogamy. Unlike the situation in *Neurospora*, however, duplicated sequences are subjected to DNA methylation only; mutations do not occur [21], [32]–[34]. Interestingly, the *Ascobolus* RID orthologue, Masc1, is essential for normal sexual development. This is in contrast to homozygous *rid* crosses, which are completely fertile [20]. It is impossible to test the involvement of Masc1 in MIP in crosses homozygous for mutations in *masc1,* but MIP is markedly reduced in crosses heterozygous for mutations in this gene (Malagnac, et al., 1997). In addition, *masc1* is more penetrant when the mutation and the duplication are in the same nucleus. These findings contrast with what has been found with *rid* mutants in *Neurospora*, which show no nucleus-specific effect [20]. Based on its involvement in MIP, Masc1 has been called a “de novo” DMT [21].

The present study was prompted by the observation that the genome of *A. nidulans* contains a single gene that is predicted to encode a DMT-like enzyme similar to Masc1 and RID. This was surprising, given that neither widespread DNA methylation nor active MIP or RIP have been reported in any *Aspergillus* species. We therefore cloned and characterized the *Aspergillus* homologue and named it *DNA methyltransferase homologue A* (*dmtA*). Curiously, the *dmtA* locus encodes both a sense (*dmtA*) and an anti-sense transcript (*tmdA*). Inactivation of *dmtA*, but not of *tmdA*, abolishes the formation of viable ascospores. No cytosine methylation has been detected in the wild type or *dmtA/tmdA* mutant strains in sexual or asexual tissues.

## Materials and Methods

### Bacterial strains and plasmids construction

*Escherichia coli* K12 XL1-Blue MR (Stratagene, La Jolla, CA, USA) was the host for most plasmid DNA. When non-methylated DNA was needed for enzyme digestions, either GM2163 or JM110 [35] were used. Plasmid pDC1 was described in Aramayo *et al*. [36]. Plasmid pRB2 was provided by Thomas H. Adams (Monsanto, St. Louis, MO). Oligonucleotides used in this study are described in Table 1.

**Table 1:** Oligonucleotides used in this study

Name	Sequence
ANID1	5′-(4110)-GGCGGTGGGGACAATTATCATCCCTCT-(4136)-3′
ANID2	5′-(4334)-TCATTGGTTTTCGTGCAATTCACGCTC-(4308)-3′
ODLAM008	5′-(2957)-CAAAGACCACCCAGACAAAT-(2976)-3′
ODLAM009	5′-(4229)-CCTCTTTACCTGCGTCCTAC-(4210)-3′
ODLAM069	5′-GGATCC-(4461)-GAGCCTCTTCCCGTTGTCCA-(4442)-3′
ODLAM070	5′-(5045)-CAGGTCTCCCCGCAATCTCC-(5064)-3′
ODLAM087	5′-CTCGAG-(2334)-ACTGACCAGCCTTTTCTCCTCGTA-(2357)-3′
ODLAM088	5′-GGTACC-(4547)-CAATCAACCGCTTACACAGACCAG-(4524)-3′

Numbers in parenthesis denote *dmtA/tmdA* coordinates with respect to the *Eco*RI site 2,432 bp upstream of the predicted translation initiation codon (ATG) of DmtA. *Bam*HI, *Xho*I, and *Kpn*I sites in ODLAM069, ODLAM087 and ODLAM088, respectively, are underlined.

**Figure 1:**
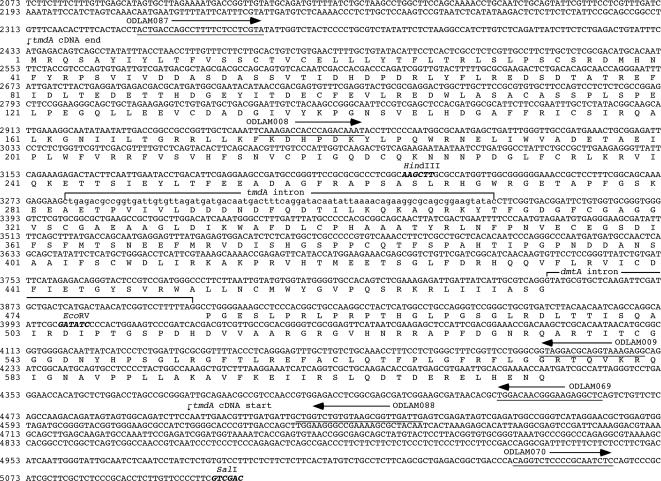
Molecular structure of the *dmtA/tmdA* chromosomal locus. The positions of the main restriction sites present in the region as well as the names, location and orientation of the oligonucleotides used are indicated above the DNA sequence. The DNA sequence of the oligonucleotides used is underlined. The 5′- and 3′-ends of the sequenced cDNAs corresponding to the *tmdA* transcript along with the *tmdA* intron (lower case) and the predicted *dmtA* intron, are indicated above the DNA sequence. The region from *Hin*dIII (coordinate 3224) to *Eco*RV (coordinate 3999) was deleted to generate the *Δ(3224–3999)dmtA* ^−^ */tmdA* ^−^ *::argB* ^+^ allele. Similarly, the sequence from the 5′-most base of oligonucleotide ODLAM069 (coordinate “4462”) to *Sal*I (coordinate 5107) was deleted to generate the *Δ(4462–5107)dmtA* ^−^ */tmdA* ^−^ *::argB* ^+^ allele. The region from ODLAM087 to ODLAM088 was fused to the *niiA* promoter to generate the *niiA(p)::dmtA* ^+^[2334–4547]*::argB* ^+^ allele. See text for details.

Plasmids were constructed by standard procedures [37]. The *Eco*RI site 2432 bp upstream of the predicted translation initiation signal (ATG) of the DmtA polypeptide was arbitrarily defined as position 1 of the *dmtA/tmdA* locus (see Figure 1). To obtain plasmid pMF156, we used the sequence information provided by the EST sequences identified in the University of Oklahoma *A. nidulans* cDNA database to design oligonucleotides (AND1 and AND2, Table 1) that were used to amplify a genomic DNA fragment from a wild-type *A. nidulans* strain (FGSC 4, Table 2). This amplified fragment was inserted into the TA-cloning vector pCR2.1 (Invitrogen) to yield pMF156. Plasmid pDLAM001 was obtained from a phage Lambda ZAP cDNA library (as described in Results) and contains the 1989 bp *tmdA* cDNA (coordinates 4503 to 2433, Figure 1). Plasmid pDLAM002 was constructed by replacing the 694 bp *Hin*dIII-*Eco*RV (coordinates 3224 to 3999) region of *dmtA/tmdA* of pDLAM001 with the ∼1.8 kbp *Nru*I-*Sma*I fragment containing the *argB* ^+^ from pDC1 after subjecting *Hin*dIII-*Eco*RV-digested pDLAM001 to an end-filling reaction. The *dmtA* and *argB* genes have opposite directions of transcription and the region deleted contains the predicted catalytic site of the putative DNA methyltransferase. Plasmids pDLAM004 and pDLAM005 contain the 3224 bp *Eco*RI-*Hin*dIII fragment of *dmt*A (coordinates 1 to 3224) and 2972 bp *Hin*dIII-*Eco*RI fragment of *dmt*A (coordinates 3224 to 6195), respectively. Insertion of the 2972 bp *Hin*dIII-*Eco*RI fragment of *dmtA/tmdA* (coordinates 3224 to 6195) into the *Hin*dIII-*Eco*RI sites of pK19 [38] yielded pDLAM013. Inverse PCR with ODLAM069, which introduces a *Bam*HI site, and ODLAM070 as primers and pDLAM013 as the substrate, followed by self-ligation resulted in pDLAM014. Plasmid pDLAM018 was constructed by inserting the ∼1.8 kbp *Bam*HI-*Xho*I fragment containing the *argB* ^+^ gene from pDC1 into the *Bam*HI-*Sal*I sites of pDLAM014, resulting in a 646 bp deletion (coordinates 4462 to 5107) of the *tmdA* promoter. Insertion of the 1226 bp *Cla*I-*Hin*dIII *dmtA/tmdA* fragment (coordinates 1998 to 3224) from pDLAM004 into the *Cla*I-*Hin*dIII sites of pDLAM005 yielded pDLAM039, which carries the 4197 bp *Cla*I-*Eco*RI *dmtA/tmdA* fragment (coordinates 1998 to 6195) inserted into the *Cla*I-*Eco*RI sites of pBluescript II KS(+) (Stratagene, La Jolla, CA, USA). Amplification of the 2214 bp fragment (coordinates 2334 to 4547) with oligonucleotides ODLAM87 and ODLAM088 as primers and pDLAM039 as substrate, followed by digestion with *Xho*I and *Kpn*I and insertion into the *Xho*I-*Kpn*I sites of pBluescript II KS(+) resulted in pDLAM040. An over-expression construct (pDLAM044) was generated by insertion of the 2214 bp *Xho*I-*Kpn*I fragment (coordinates 2334 to 4547) from pDLAM040 into the *Xho*I-*Kpn*I sites of pRB2, followed by insertion of the *niiA* promoter [39] to direct transcription of *dmtA*. These constructs carried the entire *niiA-niaD* intergenic region.

### *Aspergillus* strains and culture conditions

Strains of *A. nidulans* are described in Table 2. Standard conditions were used to maintain and grow cultures, and fungal transformations, fertility tests and genetic crosses were performed according to published protocols [40]–[43]. Minimal medium was prepared as described (Pontecorvo *et al.* 1953) with minor modifications (6 g/L of NaNO_3_). Self-fertilization assays were performed by inoculating ∼10^8^ conidia onto properly supplemented solid minimal medium (40 ml/Petri dish). To induce sexual development, we restricted air and light for 24 h after inoculation [44]. Petri dishes were incubated in the dark at 37°C for an additional 20 days, after which sexual structures (cleistothecia) could readily be observed.

**Table 2:** *Aspergillus nidulans* strains used in this study

Name^a^	Genotype^b^, ^ c^	Origin^d^
FGSC 4		FGSC^e^
FGSC A237	*pabaA1, yA2; veA1, trpC801*	FGSC^e^
FGSC A851	*pabaA1, yA2; ΔargB::trpC* ^+^ *; veA1, trpC801*	FGSC^e^
PW1	*biA1; argB2; methG1; veA1*	P. Weglenski. Department of Genetics. Warsaw University, Poland
DLAN2	*pabaA1, yA2, Δ(3224–3999)dmtA* ^−^/*tmdA* ^−^ *::argB* ^+^ *; ΔargB::trpC* ^+^ *; veA1, trpC801*	FGSC A851 transformed with pDLAM002
DLAN3	*pabaA1, yA2, Δ(4462–5107)dmtA* ^+^/*tmdA* ^−^ *::argB* ^+^ *; ΔargB::trpC* ^+^ *; veA1, trpC801*	FGSC A851 transformed with pDLAM018
DLAN4	*pabaA1, yA2; ΔargB::trpC* ^+^ *; veA1, trpC801; niiA(p)::dmtA* ^+^[2334–4547]*::argB* ^+^ [ectopic]	FGSC A851 transformed with pDLAM044
DLAN5	*biA1; methG1; veA1*	Cross of FGSC A237 with PW1
DLAN6	*pabaA1, yA2, Δ(3224–3999)dmtA* ^−^/*tmdA* ^−^ *::argB* ^+^ *; veA1*	Progeny from DLAN2 X DLAN5
DLAN7	*pabaA1, yA2, Δ(4462–5107)dmtA* ^+^/*tmdA* ^−^ *::argB* ^+^ *; veA1*	Progeny from DLAN3 X DLAN5
DLAN8	*pabaA1, yA2; veA1*	Progeny from FGSC A851 X PW1

a DLAN indicate strains constructed for this study by Dong W. Lee.

b Allele numbers or designations are: *arginine requirement-B* (ornithine transcarbamylase), *argB*; *biotin requirement-A*, *biA1*; *DNA methyltransferase-like-A* anti-sense, non-coding transcript, *tmdA*; *DNA methyltransferase-like-A*, *dmtA*; *methionine requirement-G1* (cystathionine-β-lyase), *methG1*; *nitrite utilization* (nitrite reductase), *niiA*; *p-aminobenzoic acid requirement-A1*, *pabaA1*; *tryptophan requirement-C* (IGP-synthetase, PRA-isomerase, anthranilate-synthetase, phosphorybosylanthranilate-isomerase), *trpC801*; *velvet-A1*, *veA1*; *yellow conidia-A2* (laccase-I), *yA2*.

c Note that the presence of *argB* ^+^ *or ΔargB::trpC* ^+^ *;* and *trpC* ^+^ *or trpC801* alleles was not determined in strains DLAN6, DLAN7 and DLAN8.

d Construction of the different plasmids is described in Materials and Methods.

e FGSC, indicates strains acquired from the Fungal Genetics Stock Center, University of Kansas Medical Center, Kansas City.

### DNA isolation

DNA extractions from *A. nidulans* were performed as described previously for *N. crassa* [45]. Procedures for Southern blot analysis, and other nucleic acid manipulations were as described [36], [45], [46].

### Total RNA isolation

Total RNA was extracted from mycelia using Trizol (Invitrogen, Carlsbad, CA, USA). Total RNA was extracted following the manufacturer's protocol. The resulting RNA was subjected to an additional LiCl precipitation purification step to remove DNA, which consisted of adjusting the final LiCl concentration of the aqueous RNA solution to 2M (from 10M LiCl stock solution). Samples were then incubated at 68°C for 10 min (or until the LiCl dissolves and the cloudiness of the samples disappears), and at 5°C for no less than 6 hours. Samples were then centrifuged for 30 min at room temperature. The resulting RNA pellet was then washed with 70% Ethanol and re-dissolved in water.

### Strand-specific Reverse Transcribed-PCR (SSRT-PCR)

For first strand synthesis, 0.1 µg of RNA was mixed with 10 pmoles of each ODLAM008 (*tmdA*) or ODLAM009 (*dmtA*) (Table 1) to prime *tdmA* or *dmtA* transcripts, respectively, in a 10 µl reaction volume containing 50 mM Tris-HCl, pH 8.3, 10 mM DTT, 75 mM KCl, 6 mM MgCl_2_ and 1 mM dNTP's. The mixture was denatured at 72°C for 3 min. The Reverse Transcriptase Superscript II (Invitrogen) was added (200 Units) and the reaction was incubated at 42°C for 30 min. The reaction was stopped by heating at 72°C for 30 min. Amplification of the cDNA was performed by mixing 2.5 µl of the first strand synthesis reaction product (cDNA) with 10 pmoles of ODLAM008 and ODLAM009 in a 50 µl reaction containing 40 mM Tricine-KOH (pH 8.7), 15 mM potassium acetate, 3.5 mM magnesium acetate, 3.75 µg/ml bovine serum albumin, 0.005% Tween-20, 0.005% Nonidet-P40, 0.2 mM dNTPs and 1 X polymerase mix (Advantage 2; Clontech) and subjecting reactions to the following temperature cycles: 1 cycle at 95°C for 5 min; 30 cycles at 94°C for 10 sec, 60°C for 15 sec and 68°C for 1 min. The last cycle was followed by a 2 min extension at 68°C. A fraction (10%) of the amplified mixture was gel-fractionated in 1% agarose and transferred to nylon membrane (Nytran; Schleicher & Schuell). Blots were hybridized with radiolabeled DNA fragments corresponding to the 1989 bp *Eco*RI-*Xho*I fragment (coordinates 2433 to 4503) from pDLAM001 as probe.

### Treatment of mycelia with 5-azacytidine (5-AC)

Conidia were propagated in liquid medium containing different concentrations of 5-AC for 72 hr essentially as described previously by Tamame *et al.* [47]. Conidiation of the resulting mycelial mass was induced as described [46] and the resulting conidia were diluted and spread onto Petri plates. The frequency of “fluffy” phenotypes obtained from the different strains tested was then determined among the different 5-AC concentrations tested.

### GenBank accession numbers

The combined sequence of the *dmtA/tmdA* inserts contained in pDLAM004 and pDLAM005 was deposited as GenBank accession number AF428247. Accession numbers for predicted fungal DmtAs are: Ao, *A. oryzae* (BAE61916); Af, *A. fumigatus* (XP_747703); At, *A. terreus* (XP_001209776)*;* Ci, *C. immitis* (XP_001239116); Ur, *Unicocarpus reesii* (UREG_03572.1, Broad Institute); Nc, *N. crassa* (AAM27408); Nt, *N. tetrasperma* (AAM27410); Ni, *N. intermedia* (AAM27409); Cg, *C. globosum* (XP_001222613); Gz, *G. zeae* (XP_388824); Nh, *N. haematococca* (e.gw1.11744.1, DOE); Mg, *M. grisea* (XP_366719); Bf, *B. fuckeliana* (BC1G_09864.1; BC1G_00725.1, Broad Institute); Ss, *S. sclerotiorum* (SS1G_05055.1; SS1G_00377.1, Broad Institute); and Ai, *A. immersus* (AAC49849).

## Results

### Cloning and structure of the *dmtA/tmdA* locus

Three Expressed Sequence Tags (ESTs; 13g06a1.f1, 13g06a1.r1 and g6e08a1.r1) with similarity to DMTs were identified in the University of Oklahoma *A. nidulans* cDNA database (http://www.genome.ou.edu/fungal.html). All belonged to a single gene that we named *DNA methyltransferase-like A* (*dmtA*). A gel-purified DNA fragment obtained from plasmid pMF156 was used to screen a pWE15-based *Aspergillus* cosmid library (see http://www.fgsc.net/nidlib.html) by colony hybridization. We obtained seven overlapping clones, but all were rearranged and contained only part of the *dmtA* gene. We therefore screened a non-amplified 24 h developmental phage Lambda ZAP cDNA library (see http://www.fgsc.net/nidlib.html) with a 0.5 kbp fragment containing sequences predicted to encode the amino terminus of the putative cytosine methyltransferase. Four independent clones were isolated, converted to plasmid DNA [48], sequenced and found to be identical (pDLAM001). Surprisingly, based on the directional cloning strategy used in the construction of the cDNA library, the insert present in pDLAM001 represented anti-sense transcripts (see Figure 1). The anti-sense message of *dmtA* was named *tmdA.*

To isolate and characterize the complete genomic version of the *dmtA/tmdA* locus, we constructed an *Eco*RI-*Hin*dIII mini-genomic library of the wild-type *A. nidulans* strain FGSC 4 (Table 2) by inserting genomic DNA into pBluescript II KS(+) digested with *Eco*RI and *Hin*dIII. We screened this library by colony hybridization with a labeled *dmtA/tmdA* probe from pDLAM001 and found two positive colonies. Sequencing of the corresponding plasmids (pDLAM004 and pDLAM005) showed that they span the entire *dmtA/tmdA* locus, which maps to contig 110 (195304-197205[+]) of the *A. nidulans* genome (http://www.broad.mit.edu/annotation/genome/aspergillus_nidulans/Home.html) on Chromosome 1.

The *dmtA/tmdA* chromosomal region contains one large open reading frame (*dmtA* ORF, AN6638.2; predicted translation initiation signal at coordinate 2433 in Figure 1) interrupted by a single 56 bp intron with canonical 5′- and 3′-consensus splice sites (positions 3851 and 3906, respectively). Conceptual translation of the *dmtA* ORF predicts a 615 amino acid (aa) polypeptide. Interestingly, analysis of the sequence corresponding to the *tmdA* cDNA revealed the presence of an 82 bp intron with canonical 5′- and 3′-consensus splice sites, at positions 3280 and 3361, respectively. No long ORFs were found in the *tmdA* cDNA when searching with a window of 100 aa suggesting that the *tmdA* transcript is non-coding RNA.

**Figure 2:**
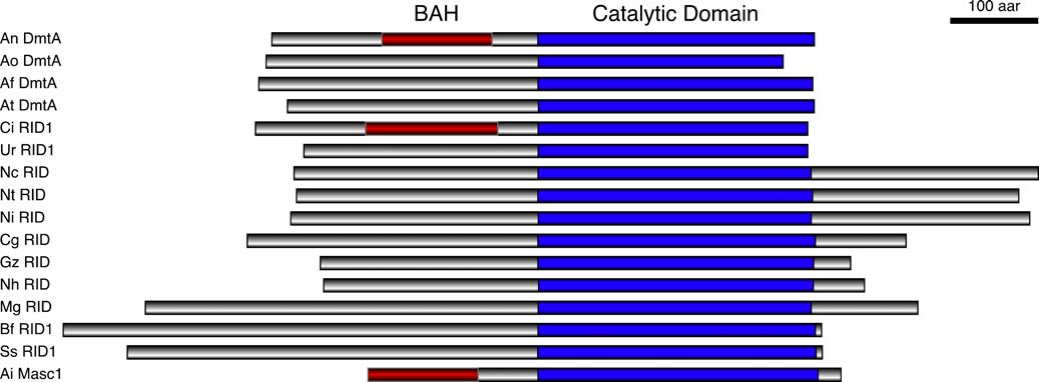
Structure of DmtA. Domain structure of DmtA and its known homologues from filamentous fungi. Confirmed and predicted proteins were aligned at the beginning of the predicted DMT catalytic domain [blue box; [77]]. Weak homology to a bromo-adjacent homology domain (BAH; red box) can be detected in DmtA homologues from *Aspergillus*, *Coccidioides* and *Ascobolus*, but is absent from the other proteins depicted. Note the long carboxy-terminal extension of *Neurospora*, and to a lesser degree *Chaetomium*, *Magnaporthe, Gibberella*, and *Nectria* Masc1/RID proteins. Abbreviations are: An, *A. nidulans*; Ao, *A. oryzae*; Af, *A. fumigatus*; At, *A. terreus;* Ci, *C. immitis*; Ur, *Unicocarpus reesii*; Nc, *N. crassa*; Nt, *N. tetrasperma*; Ni, *N. intermedia*; Cg, *C. globosum*; Gz, *G. zeae*; Nh, *N. haematococca*; Mg, *M. grisea*; Bf, *B. fuckeliana*; Ss, *S. sclerotiorum*; Ai, *A. immersus*.

BlastP searches [49] with the predicted DmtA sequence as bait revealed similarities with the group of DMT homologues found to date only in fungi and predominantly in the Ascomycota (with the notable exception of the Saccharomycotina) (Figure 2). Similarity of DmtA to its homologues in *Aspergillus oryzae* (61.5% identity), *Aspergillus fumigatus* (69.4% identity), and *Aspergillus terreus* (59.9% identity) extends across their entire predicted polypeptides. Among characterized proteins, DmtA is most similar to *N. crassa* RID (41.4% identity) [20] and *A. immersus* Masc1 (43.3% identity) [21], the putative DMTs involved in RIP and MIP, respectively. Conserved regions are restricted predominantly to the ∼300 aa catalytic DMT domain. Like other putative DMTs in the Masc1/RID family, DmtA has conserved DMT domain motifs arranged as in most eukaryotic and prokaryotic DMTs 50, 51 and it has a short variable region between motifs VIII and IX (Figure S1). When the sequence of the DmtA polypeptide was scanned against the Conserved Domain Database (CDD) using an E-value threshold of 1.0, we found a Bromo-Adjacent Homology motif (BAH; residues 136 to 236). BAH motifs are also found in *Ascobolus* Masc1 and the *Coccidioidis immitis* homologue CIMG_10138.2, but they are absent from most of these putative DMTs. Curiously, we found two RID-like proteins in the genome of the Hymenomycete *Coprinopsis cinerea* (i.e., CC1G_01237.2 and CC1G_00579.1) but both have interrupted catalytic domains and only one (CC1G_00579.1) has a complete BAH domain (data not shown). Similarly, the genomes of *Sclerotinia sclerotiorum* (SS1G_05055.1 and SS1G_00377.1) and *Botryotinia fuckeliana* (BC1G_09864.1 and BC1G_00725.1) encode two putative RID homologues. Like Ascobolus Masc1, DmtA homologues from all *Aspergillus* species, *Coccidioides, Uncinocarpus, Botryotinia,* and *Sclerotinia* lack a carboxy-terminal domain after the catalytic domain. This C-terminal region is most prominent in *Neurospora* species (Freitag et al., 2002), but shorter versions are also found in *Chaetomium, Giberella, Nectria,* and *Magnoporthe.*

### The *dmtA/tmdA* locus is not essential

**Figure 3:**
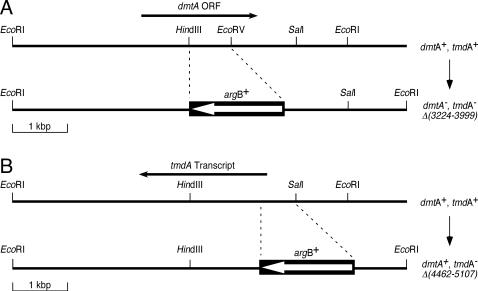
Construction of mutant alleles of the *dmtA/tmdA* region. Panels A and B show the segments of the *dmtA/tmdA* region and the relative positions of relevant restriction sites. (A) Extent of the deletion in *Δ(3224–3999)dmtA* ^−^/*tmdA* ^−^ *::argB* ^+^ allele. (B) Replacement of the *tmdA* promoter with *argB* ^+^ in *Δ(4462–5107)dmtA* ^+^/*tmdA* ^−^ *::argB* ^+^ allele.

To investigate the possible biological function of the *dmtA/tmdA* region, we deleted a 775 bp fragment within the predicted coding region of *dmtA* and replaced it with the *argB* ^+^ gene. The deleted DNA fragment contained most of the predicted catalytic domain of DmtA and included a part of the *tmdA* transcript. To do this, we transformed *A. nidulans* strain FGSC A851 with linearized pDLAM002, and selected for growth on medium without arginine (Figure 3A), thereby generating strain DLAN2 (*dmtA* ^−^/*tmdA* ^−^). We also evaluated the biological relevance of *tmdA* by deleting a 645 bp fragment corresponding to its predicted promoter, and by replacing it with the *argB* ^+^ marker from pDLAM018 (Figure 3B) in strain FGSC A851 to generate strain DLAN3 (*dmtA* ^+^/*tmdA* ^−^). Although we had to search through more than 30 strains to find the desired replacements in each case, the fact that we were able build the strains implies that the *dmtA/tmdA* locus does not encode essential gene products. Morphological phenotypes were not detected on these mutants during vegetative growth.

### Transcripts originating from the *dmtA/tmdA* locus are scarce and constitutively produced

**Figure 4:**
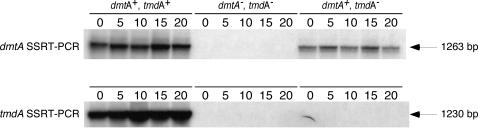
Transcription of the *dmtA/tmdA* locus. Developmental regulation of the *dmtA/tmdA* locus. Southern blot of DNA synthesized by a strand-specific reverse transcribed-PCR reaction (SSRT-PCR), with mRNA extracted from strains FGSC A851 (*dmtA* ^+^ */tmdA* ^+^), DLAN2 (*dmtA* ^−^ */tmdA* ^−^), and DLAN3 (*dmtA* ^+^ */tmdA* ^−^), at 0, 5, 10, 15, and 20 hours after synchronously inducing asexual development [78]. For details, see Materials and Methods (Strand-specific Reverse Transcribed-PCR (SSRT-PCR)).

No *dmtA* or *tmdA* transcripts were detectable by Northern blot hybridizations using 50 µg of total RNA extracted from vegetative mycelium and self-fertilized fruiting bodies of a wild type strain (data not shown). However, both *dmtA* and *tmdA* transcripts were detectable by Strand-Specific Reverse Transcribed-PCR in mRNA from wild-type vegetative cells (Figure 4). Results from these experiments suggested that the *dmtA/tmdA* region is constitutively transcribed on both strands during asexual and sexual development (Figure 4 and data not shown). The anti-sense *tmdA* transcripts appear slightly more abundant than those of *dmtA* (Figure 4), which may be why all ESTs sequenced from this region correspond to *tmdA*. As expected, neither *dmtA* nor *tmdA* transcripts were detected in the *dmtA* ^−^/*tmdA* ^−^ DLAN2 strain (Figure 4).

The existence of non-coding anti-sense transcripts in the *dmtA/tmdA* region suggested the possibility of functional interactions between the sense and anti-sense transcripts. In one such model, antisense transcripts may serve to regulate the level of DmtA protein by forming double-stranded RNA (dsRNA), which then would be expected to serve as a target for ribonucleases like Dicer [52], [53]. If this were the case, our inability to readily detect transcripts by standard Northern blot analysis might reflect rapid degradation, e.g. into small-interfering RNAs (i.e., siRNAs). However, we found that disruption of the promoter controlling the production of *tmdA* transcript in strain DLAN3 (*dmtA* ^+^/*tmdA* ^−^) failed to increase the level of *dmtA* transcripts significantly, arguing against this scenario (Figure 4 and data not shown). We also sought to perturb potential dsRNA formation by increasing *dmtA* transcript levels in *trans*. For this, we over-expressed *dmtA* by constructing a transcriptional fusion between the inducible *niiA* (nitrite reductase) promoter [39], and the *dmtA* coding region. The resulting plasmid (pDLAM044) was linearized and used to transform strain FGSC A851. Transformants were selected on minimal medium supplemented with *p*-aminobenzoic acid and strains with a single ectopic copy of the *niiA*(p)::*dmtA* ^+^::*argB* ^+^ fusion construct were identified by Southern hybridization. Over-expression of *dmtA* ^+^ in DLAN4 (*niiA(p)::dmtA* ^+^) strain was confirmed by Northern analysis and did not result in RNA degradation or other noticeable phenotypes (data not shown).

### *dmtA* is dispensable for asexual development but essential for sexual development

We tested if *dmtA* ^−^/*tmdA* ^−^ (DLAN2), *dmtA* ^+^/*tmdA* ^−^ (DLAN3) and *niiA*(p)::*dmtA* ^+^; *dmtA* ^+^/*tmdA* ^+^ (DLAN4) strains were defective in the development and formation of asexual reproductive structures or conidiophores, and if the resulting asexual spores (i.e., conidia) could germinate normally. In all mutants, we found that asexual development, spore formation and spore germination were indistinguishable from those of wild-type strains.

**Figure 5:**
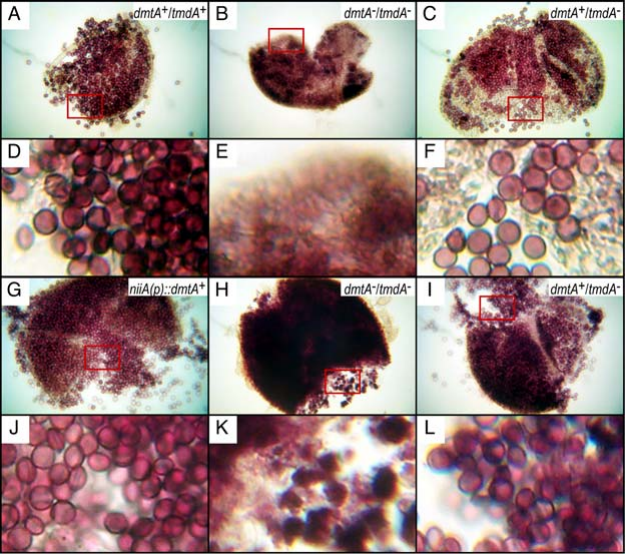
Sexual development is abolished in *dmtA* ^−^ */tmdA* ^−^ mutants but unaffected in *dmtA* ^+^ */tmdA* ^−^ mutants and *niiA(p)::dmtA* ^+^ transformants. Panels A, B, C, G, H and I show representative cleistothecia from self-fertilized wild type *dmtA* ^+^/*tmdA^+^* (FGSC A851), the *dmtA* ^−^/*tmdA* ^−^ mutant (DLAN2), the *dmtA* ^+^/*tmdA* ^−^ mutant (DLAN3), the DmtA overexpressing *niiA(p)::dmtA* ^+^[2334–4547] transformant (DLAN4), and the back-crossed *dmtA* ^−^/*tmdA* ^−^ (DLAN6) and *dmtA* ^+^/*tmdA* ^−^ mutants (DLAN7), respectively. Cleistothecia were obtained as described in Materials and Methods, cleaned, crushed and photographed using a Zeiss light microscope at 400X magnification. The area highlighted by the rectangles in Panels A, B, C, G, H, and I, was enlarged six-times and presented in Panels D, E, F J, K, and L, respectively.

To determine if *dmtA* or *tmdA* are required for sexual development, we self-fertilized strains DLAN2 and DLAN3 and compared the crosses to those of wild-type strains. Interestingly, a *dmtA* ^−^/*tmdA* ^−^ mutant (DLAN2) formed exclusively immature fruiting bodies without ascospores (Figures 5B and 5E) whereas control crosses of wild-type strains produced normal fruiting bodies with abundant sexual spores (Figures 5A and 5D). In contrast, the sexual development of the *dmtA* ^+^/*tmdA* ^−^ (DLAN3) mutant was indistinguishable from that of the wild type (Figures 5C and 5F).

To ensure that the phenotype observed in *dmtA* ^−^/*tmdA* ^−^ disruption strains was not caused by an unlinked mutation introduced during transformation, both DLAN2 and DLAN3 strains were crossed to DLAN5 (Table 2). Ascospores from these backcrosses were germinated and a total of 24 recombinants (12 per cross) carrying either the *dmtA* ^−^/*tmdA* ^−^ or the *dmtA* ^+^/*tmdA* ^−^ allele, were selected and tested for their ability to undergo homothallic sexual development. Results from this experiment were consistent with our previous observation. All 12 *dmtA* ^−^/*tmdA* ^−^ progeny (from the *dmtA* ^−^/*tmdA* ^−^ x *dmtA* ^+^/*tmdA* ^+^ cross) were sterile (DLAN6. Figures 5H and 5K—these figures show the presence of maternal tissue exclusively), whereas the 12 *dmtA* ^+^/*tmdA* ^−^ progeny (from the *dmtA* ^+^/*tmdA* ^−^ x *dmtA* ^+^/*tmdA* ^+^ cross) were fertile (DLAN7. Figures 5I and 5L). Another control cross (FGSC A851 X PW1, Table 2), yielded four recombinants with the same genotype as FGSC A851, and as expected, all these strains were fertile and yielded a normal number of ascospores (data not shown). We conclude that strains lacking *dmtA* transcript are unable to complete normal sexual development.

We also observed that when self-fertilized and tested for sexual development, cultures of strains carrying the *niiA*(p)::*dmtA* ^+^; *dmtA* ^+^/*tmdA* ^+^ (DLAN4) alleles formed normal fruiting bodies with the expected abundance of fully developed and viable ascospores (Figures 5G and 5J). Together, these results suggest that the absence of *dmtA* transcript results in a pronounced defect in early sexual development.

### Search for DNA methylation in *dmtA/tmdA* mutants

To test the possible effects of *dmtA* and *tmdA* mutations, or over-expression of *dmtA,* on DNA methylation, we performed two types of experiments. We analyzed genomic DNA of wild type and mutant strains for methylation by traditional Southern hybridization as well as by immunoblotting with a sensitive monoclonal antibody against 5-methylcytosine (5Me-C; methods described in [54]. We tested DNA from both vegetative and sexual tissues (cleistothecia) of DLAN2 (*dmtA* ^−^ */tmdA* ^−^), DLAN3 (*dmtA* ^+^ */tmdA* ^−^), FGSC 4 (*dmtA* ^+^ */tmdA* ^+^) and vegetative tissues of FGSC A851 (*dmtA* ^+^ */tmdA* ^+^) and DLAN4 (*niiA*(p)::*dmtA* ^+^; *dmtA* ^+^/*tmdA* ^+^). DNA from vegetative tissue of a *N. crassa* wild type (*dim-2^+^*) and of a *dim-2* mutant (which lacks all detectable DNA methylation; [12], served as controls. For Southern hybridizations, DNAs were digested with *Sau*3AI (S) or *Dpn*II (D), which differ in sensitivity to 5MeC (S is inhibited by 5MeC whereas D is not), and probed with rDNA, which is typically methylated in organisms that sport 5MeC (Figure S2). We were unable to detect DNA methylation in vegetative or sexual tissue from *Aspergillus* wild type strains by using the *Neurospora* rDNA repeat and several known methylated retrotransposon relics from *Neurospora* (Figure S2 and data not shown). We next used the *Aspergillus* rDNA intragenic spacer region as a probe but did not find DNA methylation (data not shown). No reproducible differences in band patterns between any of the *dmtA/tmdA* mutants and wild type *Aspergillus* were found. Tests with the sensitive antibody to 5MeC showed no DNA methylation in any of the *Aspergillus* strains tested, as with negative control genomic DNA from *S. cerevisiae* and the *Neurospora dim-2* mutant (data not shown). Quantitative differences in DNA methylation levels of four different *Neurospora dim* mutants previously characterized by Southern hybridizations were confirmed with the antibody assay, suggesting that small changes in DNA methylation levels are detectable by this method (data not shown).

We also tested the effect of 5-azacytidine (5-AC) on strains DLAN2 (*dmtA* ^−^ */tmdA* ^−^), DLAN3 (*dmtA* ^+^ */tmdA* ^−^), FGSC 4 (*dmtA* ^+^ */tmdA* ^+^) and FGSC A851 (*dmtA* ^+^ */tmdA* ^+^). 5-AC, a cytidine analog is known to cause extensive DNA hypomethylation and its use and mode of action in DNA methylation has been extensively documented [55]–[57]. In *Aspergillus*, low concentrations of 5-AC are known to increase the formation of “fluffy” phenotypic variants [47], [58], an effect postulated to occur through the heritable modification of a single nuclear gene, *fluffy-F1* (*FluF1*) [58]. The frequency of non-conidial (i.e., fluffy) strains obtained in our experiments although lower from what was previously reported [47], was nevertheless similar among the different strains tested, thus these experiments did not reveal any phenotypic differences between experimental and control strains (data not shown).

## Discussion

We report the cloning and characterization of the *dmtA* gene, predicted to encode a Masc1/RID DMT-like protein from *A. nidulans*, a fungus in which no DNA methylation has been demonstrated. Curiously, we found that *dmtA* is transcribed on both strands, thus leading to the designation “*dmtA/tmdA*” for the locus. In eukaryotes, transcription on both top and bottom strands is often associated with gene silencing at either the transcriptional or post-transcriptional level (e.g., via formation of siRNAs). Our results, however, are not consistent with *tmdA* playing a role in the regulation of *dmtA.* Instead, we found that the *dmtA* transcript, but not the *tmdA* transcript, is essential for normal completion of sexual development.

In *A. nidulans*, sexual reproduction occurs after asexual sporulation has stopped and results in the formation of macroscopic fruiting bodies called cleistothecia. Although the formation of ascogenous tissue is not completely understood in this organism, its development is thought to be similar to ascogenous tissue development in *Neurospora* and *Ascobolus*, where the two nuclei fuse to generate dikaryotic tissue, which then develops further to form a three-celled hook-shaped structure called the crozier [59]. The parental nuclei in the middle cell of the crozier fuse to form a diploid nucleus, which then immediately undergoes meiosis [59]. The four resulting haploid nuclei undergo a single mitosis, resulting in eight nuclei that are partitioned into eight ascospores within the developing ascus [60]. During ascospore maturation in *A. nidulans,* the nuclei undergo a second mitotic division that results in the formation of eight bright red mature binucleate ascospores. It is noteworthy that neither of these early structures can be observed in *dmtA* ^−^ */tmdA* ^−^ mutants. In *Ascobolus*, crosses homozygous for *masc1* are blocked in sexual development before crozier formation [21]. No such defect was observed in *Neurospora* crosses homozygous for *rid* [20]. The *dmtA/tmdA* deletion phenotype observed in *Aspergillus* resembles the one seen in *Ascobolus* crosses homozygous for *masc1*-cleistothecia are devoid of internal ascogenous tissue. That development of croziers and viable ascospores was never observed in our *dmtA* mutant suggests that DmtA has an important role in sexual development, as previously suggested for Masc1 [21].

It is interesting to consider why a gene known to be involved in RIP in *Neurospora* would be quite conserved and functional in an organism apparently devoid of active RIP and DNA methylation. All *Aspergillus* species examined to date (*A. nidulans*, *A. fumigatus*, *A. terreus* and *A. oryzae*) have DmtA homologues. DmtA homologues are not restricted to the *Eurotiomycetes* (e.g., *Aspergillus*), *Sordariomycetes* (e.g., *Neurospora*), or *Pezizomycetes* (e.g., *Ascobolus*), but are also present in the *Leotiomycetes* (e.g., *Sclerotinia*). Among the *Ascomycota*, they are notably absent from the *Saccharomycotina*, which also lack components of RNA silencing machinery [61], [62].

The predicted structures of DmtA homologues, although conserved, are not identical. These proteins can be grouped by the presence of a conserved BAH domain upstream of the “catalytic” domain. Another feature that distinguishes classes of DmtA homologues is the carboxy-terminal domain. Some DmtA homologues have centrally located catalytic domains (e.g., RID from *N. crassa, N. tetrasperma*, *N. intermedia*, and *Podospora anserina*), a second group has a carboxy-terminal located catalytic domain (e.g., DmtA from *A. nidulans, A. fumigatus, A. oryzae* and *A. terreus*, the two RID homologues each from *Botryotinia fuckeliana* and *Sclerotinia sclerotiorum*, Masc1 from *A. immersus*, and RID from *C. immitis and Uncinocarpus reesii*). In the remaining members, the carboxy-terminally located peptides have varied lengths (e.g., RID from *Chaetomium globosum*, *Gibberella zeae*, *Nectria haematococca* and *Magnaporthe grisea*,). Based on these comparisons it is tempting to speculate that the C-terminal “tail” of *Neurospora* and *Podospora* RID homologues might be involved in determining RIP efficiency.

Why would the *dmtA/tmdA* locus be transcribed on both strands? In general, our understanding of the interplay of sense/anti-sense transcription is very limited, especially in fungi. Genome-wide analyses of mouse transcripts suggest that anti-sense transcription is quite widespread and, contrary to an early suggestion, is not restricted to imprinted genes [63]. The ratio of sense/anti-sense transcripts in mouse cells fluctuates markedly among different tissues consistent with the hypothesis that anti-sense transcription serves as a gene regulatory mechanism during development. Interestingly, murine anti-sense transcripts tend to be poly(A)-negative and nuclear localized, similarly to what has been found among randomly selected sense/anti-sense pairs from *Arabidopsis thaliana*, which are also poly(A)-negative and nuclear localized [64]. Perhaps the presence of these sense/anti-sense dsRNA pairs and/or their polyadenylation status determines their nuclear localization in a developmentally regulated manner.

Initial evidence for the existence of sense and anti-sense dsRNA in fungi came from studies aimed at isolating meiosis-specific genes in *Schizosaccharomyces pombe* [65]. Among several meiotic expression upregulated (meu) cDNAs characterized, five lacked clear ORFs and were postulated to represent non-coding RNA. In a more recent study, the presence of long ORFs on both strands of regions of several, distantly-related fungal genomes was taken as an indication for the presence of anti-sense transcripts [66]. The authors of this study noted that, curiously, the majority of the postulated transcript pairs have no homolog in any other characterized species, similar to the situation in mouse. They also proposed that the genes involved in sense/anti-sense-relationships code for proteins that are preferentially localized to the nucleus, as suggested by the Gene Ontology terms used to annotate them. The *IME4* gene of *S. cerevisiae* exemplifies the importance of sense-anti-sense transcription in fungi. The *IME4* anti-sense transcript causes transcriptional interference, a regulatory mechanism that controls entry into meiosis [67]. Our observation that the *dmtA/tmdA* locus is actively transcribed in both directions constitutes the first demonstration of the presence of a sense/anti-sense-pair that, analogous to the *IME4* locus in yeast, controls the early stages of sexual development in *A. nidulans*.

Finally, we did not detect DNA methylation in *A. nidulans*. This is consistent with previous studies that failed to reveal DNA methylation or MIP in this organism [47], [58], [68]. Similarly, RIP has not been detected in *A. nidulans*, despite numerous studies in which RIP, if present, should have been observable even at a low level, as was reported for *Leptosphaeria maculans* [69], [70] and *P. anserina* [71]–[73]. Neverthless, it seems possible that DmtA proteins are responsible for vestiges of RIP found in the *Aspergillus* genome and perhaps for the low RIP activity reported for some other *Aspergillus* species [74]–[76]. One possibility is that in *Aspergillus de novo* DNA methylation by DmtA only occurs transiently during the sexual phase, a stage where low levels of DNA methylation are difficult to detect. If true, this methylation might not be maintained and might only rarely result in RIP. Future studies on the expression of DmtA or Masc1 in *Neurospora* may provide clues to what makes a fungus competent for efficient MIP or RIP.

## Supporting Information

Figure S1 (0.57 MB PDF)

Figure S2 (0.59 MB PDF)
