# Supplementary material for: A Cytosine Methyltransferase Homologue Is Essential for Sexual Development in Aspergillus nidulans
Source: PLoS One. 2008 Jun 25;3(6):e2531. doi: 10.1371/journal.pone.0002531 (PMC2432034; doi:10.1371/journal.pone.0002531)
Supplement: Figure S1 — (0.57 MB PDF) [file pone.0002531.s001.pdf]

# Electronic Supplementary Materials and Methods

## Figure Supplementary 1

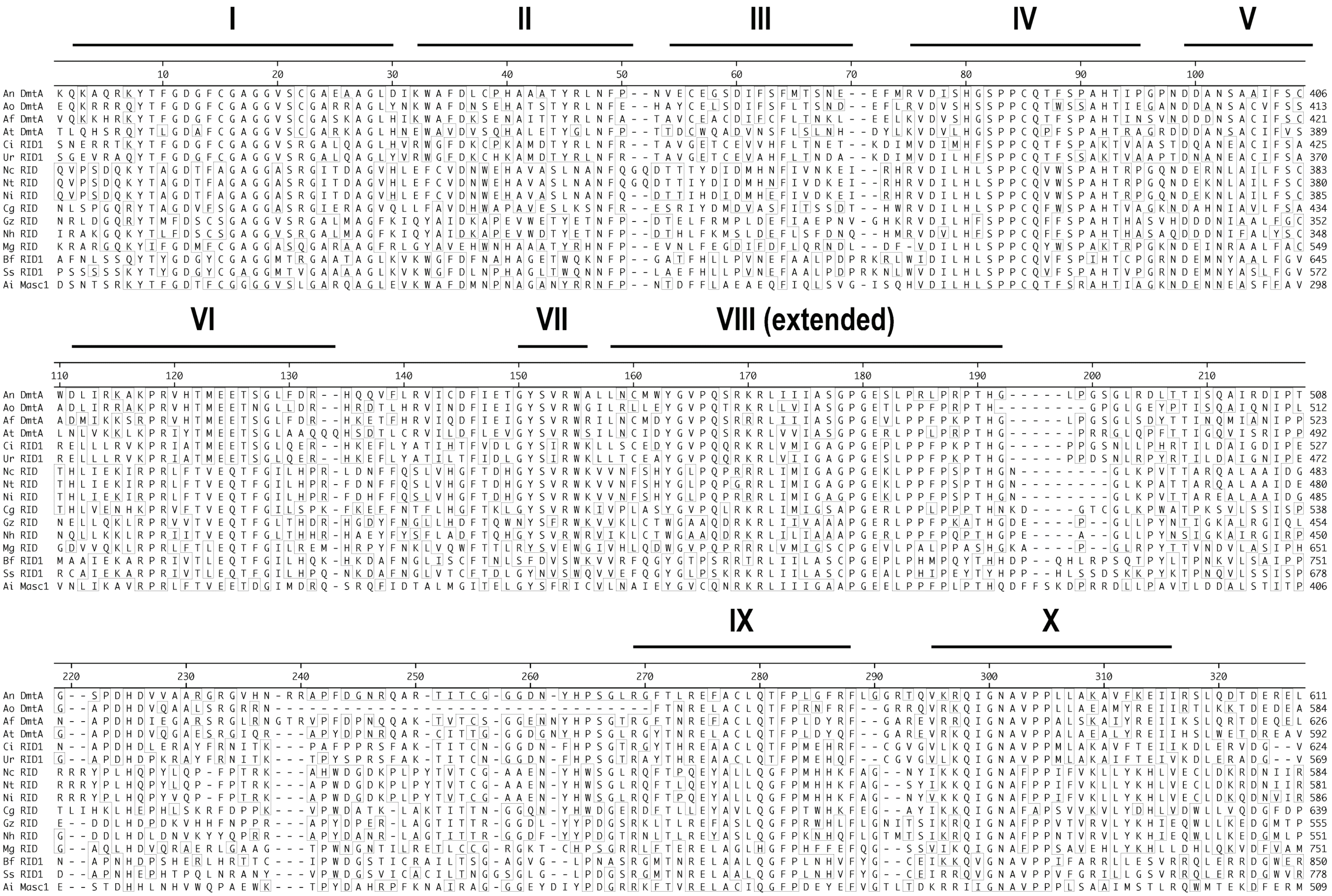
I
II
III
IV
V
VI
VII
VIII (extended)
IX
X
An DmtA
Ao DmtA
Af DmtA
At DmtA
Ci RID1
Ur RID1
Nc RID
Nt RID
Ni RID
Cg RID
Gz RID
Nh RID
Mg RID
Bf RID1
Ss RID1
Ai Masc1

**Figure S1. Alignment of the predicted catalytic domain of DmtA homologues.** Conserved DMT motifs are indicated by roman numerals. Clustal W with default settings [1] was used to align the amino acid sequences of the catalytic domains. Identical and highly conserved (i.e., present in eight or more species) are boxed together. Species abbreviations are: An, *A. nidulans*; Ao, *A. oryzae*; Af, *A. fumigatus*; At, *A. terreus;* Ci, *C. immitis*; Ur, *Unicocarpus reesii*; Nc, *N. crassa*; Nt, *N. tetrasperma*; Ni, *N. intermedia*; Cg, *C. globosum*; Gz, *G. zeae*; Nh, *N. haematococca*; Mg, *M. grisea*; Bf, *B. fuckeliana*; Ss, *S. sclerotiorum*; Ai, *A. immersus*.

**References.**

1. Thompson JD, Higgins DG, Gibson TJ (1994) CLUSTAL W: improving the sensitivity of progressive multiple sequence alignment through sequence weighting, position-specific gap penalties and weight matrix choice. Nucleic Acids Research 22: 4673-4680.
