# Supplementary material for: A Cytosine Methyltransferase Homologue Is Essential for Sexual Development in Aspergillus nidulans
Source: PLoS One. 2008 Jun 25;3(6):e2531. doi: 10.1371/journal.pone.0002531 (PMC2432034; doi:10.1371/journal.pone.0002531)
Supplement: Figure S2 — (0.59 MB PDF) [file pone.0002531.s002.pdf]

Electronic Supplementary Materials and Methods

Figure Supplementary 2

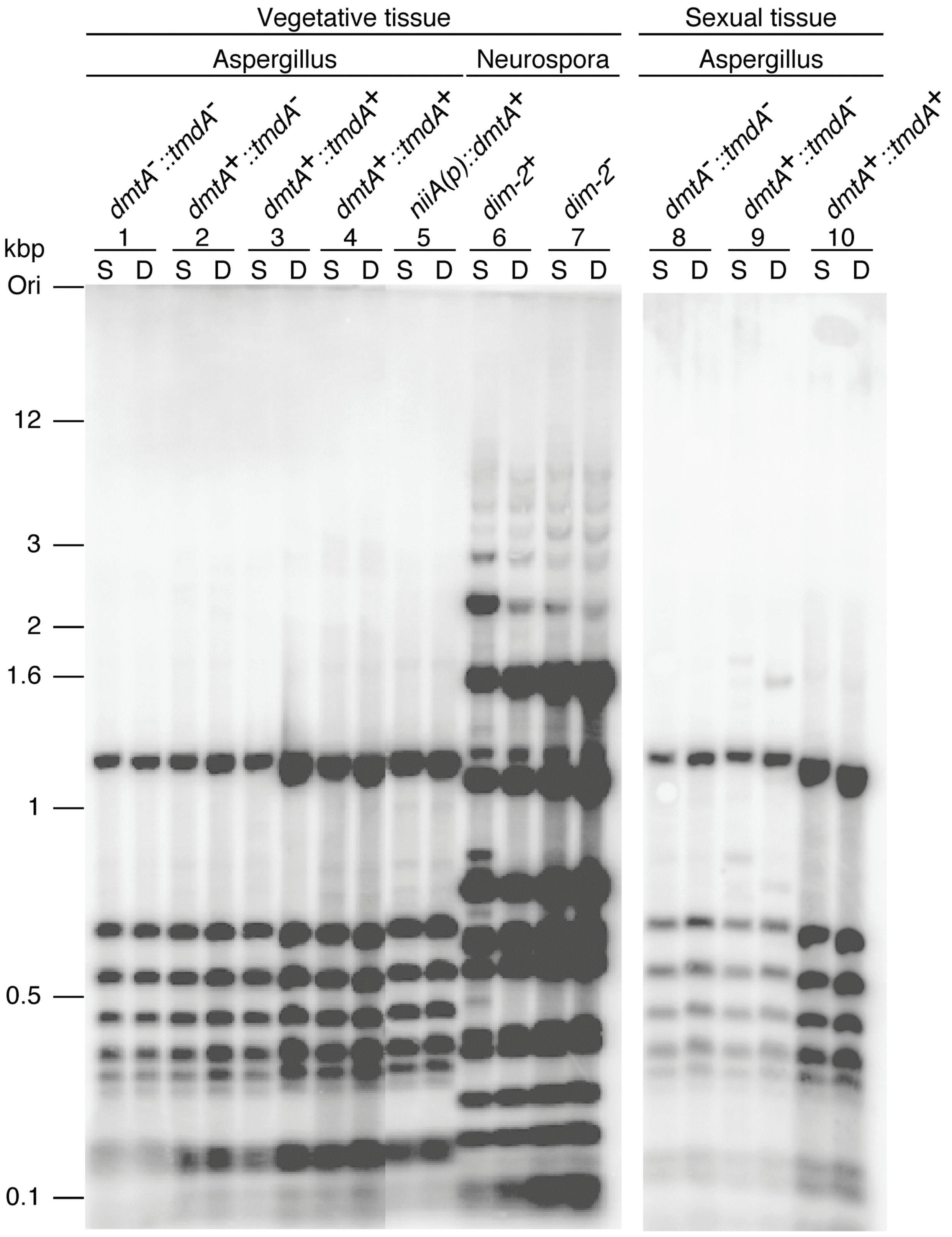

Vegetative tissue
Sexual tissue
Aspergillus
Neurospora
Aspergillus
dmtA⁻::tmdA⁻
dmtA⁺::tmdA⁻
dmtA⁺::tmdA⁺
dmtA⁺::tmdA⁺
niiA(p)::dmtA⁺
dim-2⁺
dim-2⁻
dmtA⁻::tmdA⁻
dmtA⁺::tmdA⁻
dmtA⁺::tmdA⁺
kbp
1 2 3 4 5 6 7 8 9 10
S D S D S D S D S D S D S D S D S D S D
Ori
12
3
2
1.6
1
0.5
0.1

Figure S2. No DNA methylation is detectable in *A. nidulans* by Southern analysis.

DNA was extracted from vegetative and sexual tissues (cleistothecia) of the *dmtA*$^{-}$/*tmdA*$^{-}$ mutant (DLAN2; lanes 1 and 8), the *dmtA*$^{+}$/*tmdA*$^{-}$ mutant (DLAN3; lanes 2 and 9), wild type *dmtA*$^{+}$/*tmdA*$^{+}$ (FGSC 4; lanes 3 and 10), or from vegetative tissue of *dmtA*$^{+}$/*tmdA*$^{+}$ (FGSC A851; lane 4) and the DmtA-overexpressing strain *niiA(p)::dmtA*$^{+}$[2334-4547] transformant (DLAN4; lane 5). As control, DNA was extracted from vegetative tissue of a *N. crassa* wild-type (lane 6), and a DNA methyltransferase mutant *dim-2* (lane 7). DNA was digested with *Sau*3AI (S) or with its cytosine methylation-insensitive *Dpn*II (D) isoschizomer, electrophoresed through 1% agarose gels, blotted to nylon membrane and hybridized with a radioactively labeled rDNA probe from *N. crassa*. The presence of 5MeC in the recognition sequence of these two enzymes (which is identical), will block digestion of this recognition site by S, but not by D. A restriction site that is normally methylated and not digested by S will therefore be digested by D or by the global removal of DNA methylation (e.g., by introducing a *dim-2* mutation) in the organism.
